# Age-Associated Sperm DNA Methylation Alterations: Possible Implications in Offspring Disease Susceptibility

**DOI:** 10.1371/journal.pgen.1004458

**Published:** 2014-07-10

**Authors:** Timothy G. Jenkins, Kenneth I. Aston, Christian Pflueger, Bradley R. Cairns, Douglas T. Carrell

**Affiliations:** 1 Andrology and IVF Laboratories, Department of Surgery, University of Utah School of Medicine, Salt Lake City, Utah, United States of America; 2 Department of Oncological Sciences, Huntsman Cancer Institute, University of Utah School of Medicine, Salt Lake City, Utah, United States of America; 3 Howard Hughes Medical Institute, Chevy Chase, Maryland, United States of America; 4 Department of Genetics, University of Utah School of Medicine, Salt Lake City, Utah, United States of America; 5 Department of Obstetrics and Gynecology, University of Utah School of Medicine, Salt Lake City, Utah, United States of America; Albert Einstein College of Medicine, United States of America

## Abstract

Recent evidence demonstrates a role for paternal aging on offspring disease susceptibility. It is well established that various neuropsychiatric disorders (schizophrenia, autism, etc.), trinucleotide expansion associated diseases (myotonic dystrophy, Huntington's, etc.) and even some forms of cancer have increased incidence in the offspring of older fathers. Despite strong epidemiological evidence that these alterations are more common in offspring sired by older fathers, in most cases the mechanisms that drive these processes are unclear. However, it is commonly believed that epigenetics, and specifically DNA methylation alterations, likely play a role. In this study we have investigated the impact of aging on DNA methylation in mature human sperm. Using a methylation array approach we evaluated changes to sperm DNA methylation patterns in 17 fertile donors by comparing the sperm methylome of 2 samples collected from each individual 9–19 years apart. With this design we have identified 139 regions that are significantly and consistently hypomethylated with age and 8 regions that are significantly hypermethylated with age. A representative subset of these alterations have been confirmed in an independent cohort. A total of 117 genes are associated with these regions of methylation alterations (promoter or gene body). Intriguingly, a portion of the age-related changes in sperm DNA methylation are located at genes previously associated with schizophrenia and bipolar disorder. While our data does not establish a causative relationship, it does raise the possibility that the age-associated methylation of the candidate genes that we observe in sperm might contribute to the increased incidence of neuropsychiatric and other disorders in the offspring of older males. However, further study is required to determine whether, and to what extent, a causative relationship exists.

## Author Summary

There is a striking trend of delayed parenthood in developed countries due to secular and socioeconomic pressures. As a result, physicians commonly consult with concerned patients inquiring about the impact of advanced age on their ability to conceive healthy offspring. The concern has more frequently surrounded the effects of advanced maternal age, but recent evidence suggests negative effects of advanced paternal age as well. Specifically, studies have demonstrated increased incidence of neuropsychiatric and other disorders in the offspring of older males. In this study we have investigated a commonly hypothesized mechanism for this effect, namely sperm DNA methylation alteration. Our data indicate that specific genomic regions of DNA methylation are commonly altered with age, suggesting that some regions of the sperm genome are more susceptible than others to age-related epigenetic changes. Importantly, a significant portion of these alterations occur at genes known to be associated with schizophrenia and bipolar disorder, both of which display increased incidence in the offspring of older fathers. These data will be important in driving future studies aimed at determining the impact that these methylation alterations may have on offspring health and will thus enable couples at advanced reproductive ages to be more informed of possible risks.

## Introduction

The effects of advanced paternal age have only recently become of interest to the scientific community as a whole. This interest has likely arisen as a result of recent studies that suggest an association with increased incidence of diseases and abnormalities in the offspring of older fathers. Specifically, offspring sired by older fathers have been shown to have increased incidence of neuropsychiatric disorders (autism, bipolar disorder, schizophrenia, etc.) [1]–[3], trinucleotide repeat associated diseases (myotonic dystrophy, spinocerebellar atixia, Huntington's disease, etc.) [4]–[7], as well as some forms of cancer [8]–[11]. Though these are intriguing data, we know very little about the etiology of the increased frequency of diseases in the offspring of older fathers. Among the most likely contributing factors to this phenomenon are epigenetic alterations in the sperm that can be passed on to the offspring.

These studies are in striking contrast to the previously held dogma that the mature sperm are responsible only for the safe delivery of the paternal DNA. Intriguingly, with increased investigation has come mounting evidence that the sperm epigenome is not only well suited to facilitate mature gamete function but is also competent to contribute to events in embryonic development. It has been established that even through the dramatic nuclear protein remodeling that occurs in the developing sperm, involving the replacement of histone proteins with protamines, some nucleosomes are retained [12]. Importantly, histones are retained at promoters of important genomic loci for development, suggesting that the sperm epigenome is poised to play a role in embryogenesis [12]. In addition, recent reports suggest that hypomethylated regions with high CpG density also appear to drive nucleosome retention [13]. Similarly, DNA methylation marks in the sperm have been identified that likely contribute to embryonic development as well [12], [14]. These data strongly support the hypothesis that the sperm epigenome is not only well suited to facilitate mature sperm function, but that it also contributes to events beyond fertilization.

Looking past fertilization and embryogenesis, sperm appear to contribute to events manifesting later in life. The remarkable claim that sperm, independent of gene mutation, may be capable of affecting phenotype in the offspring was initially proposed as a result of large retrospective epidemiological studies observing changes in the frequency of diseases in the offspring of fathers who were exposed to famine conditions in the early 19^th^ century [15], [16]. Recently, many studies utilizing animal models have discovered similar patterns that comport with the epidemiological data. Specifically, in male animals fed a low protein diet, offspring display altered cholesterol metabolism in hepatic tissue [17]. However, the etiology of this phenomenon is poorly understood. Despite this, there are multiple likely candidates that may drive these effects, such as DNA methylation.

Methylation marks at cytosine residues, typically found at cytosine phosphate guanine dinucleotides (CpGs), in the DNA are capable of regulatory control over gene activation or silencing. These roles are dependent on location relative to gene architecture (promoter, exon, intron, etc.). Since these heritable marks are capable of driving changes that may affect phenotype, they represent a possible mechanism to explain the increased disease susceptibility in the offspring of older fathers. Additionally, in both sexes, aging alters DNA methylation marks in most somatic tissues throughout the body. In one of the first large studies to address the question of age-associated methylation alterations, Christensen et al. identified over 300 different CpG loci with age-associated methylation alterations in many tissues [18]. One recent study compared age-associated DNA methylation alterations in blood, brain, kidney and muscle tissue and identified both common and unique methylation alterations between different tissues [19]. Additionally, recent work suggests that DNA methylation can be used to predict the age of an organism based on tissue methylation profiles [20]. This study also supports previous reports which identify global hypomethylation as a hallmark of aging in most somatic tissues [21]. Because of its prevalence in other cell types, age-associated DNA methylation alteration is likely to occur in sperm as well. In further support of this idea is work demonstrating that frequently dividing cells typically have more striking methylation changes associated with age than do cells which divide less often [22]. In this study we have analyzed the age associated sperm DNA methylation alterations that are common among the individuals in our study population to determine the magnitude of sperm DNA methylation changes over time and whether specific regions are consistently altered with age.

## Results

Our study includes 17 sperm donors (of known fertility) that collected an ejaculate in the 1990's. These donors were asked to provide an additional semen sample in 2008, enabling the evaluation of intra-individual changes to sperm DNA methylation with age. These samples are referred to as young (1990's collection) and aged (2008 collection) respectively. The age difference between each collection varied between 9 and 19 years, and the age at first collection (“young” sample) was between 23 and 56 years of age. Table 1 describes the donor demographics within both categories.

**Table 1:** Donor demographics.

Parameter	Young *(±SEM)*	Aged *(±SEM)*	Significance
**Age**	37.7 (±2.12)	50.3 (±2.1)	N/A
**Volume**	3.78 (±0.46)	2.85 (±0.45)	p = 0.0142
**Million/ml**	125.4 (±9.16)	145.56 (±15.57)	P>0.05
**Total count**	434.32 (±53.67)	424.67 (±88.69)	P>0.05
**Total motile**	63.38 (±1.64)	61.25 (±4.34)	P>0.05
**% live**	69.08 (±1.47)	61.0 (±3.93)	P>0.05

### Global methylation analysis

**Figure 1:**
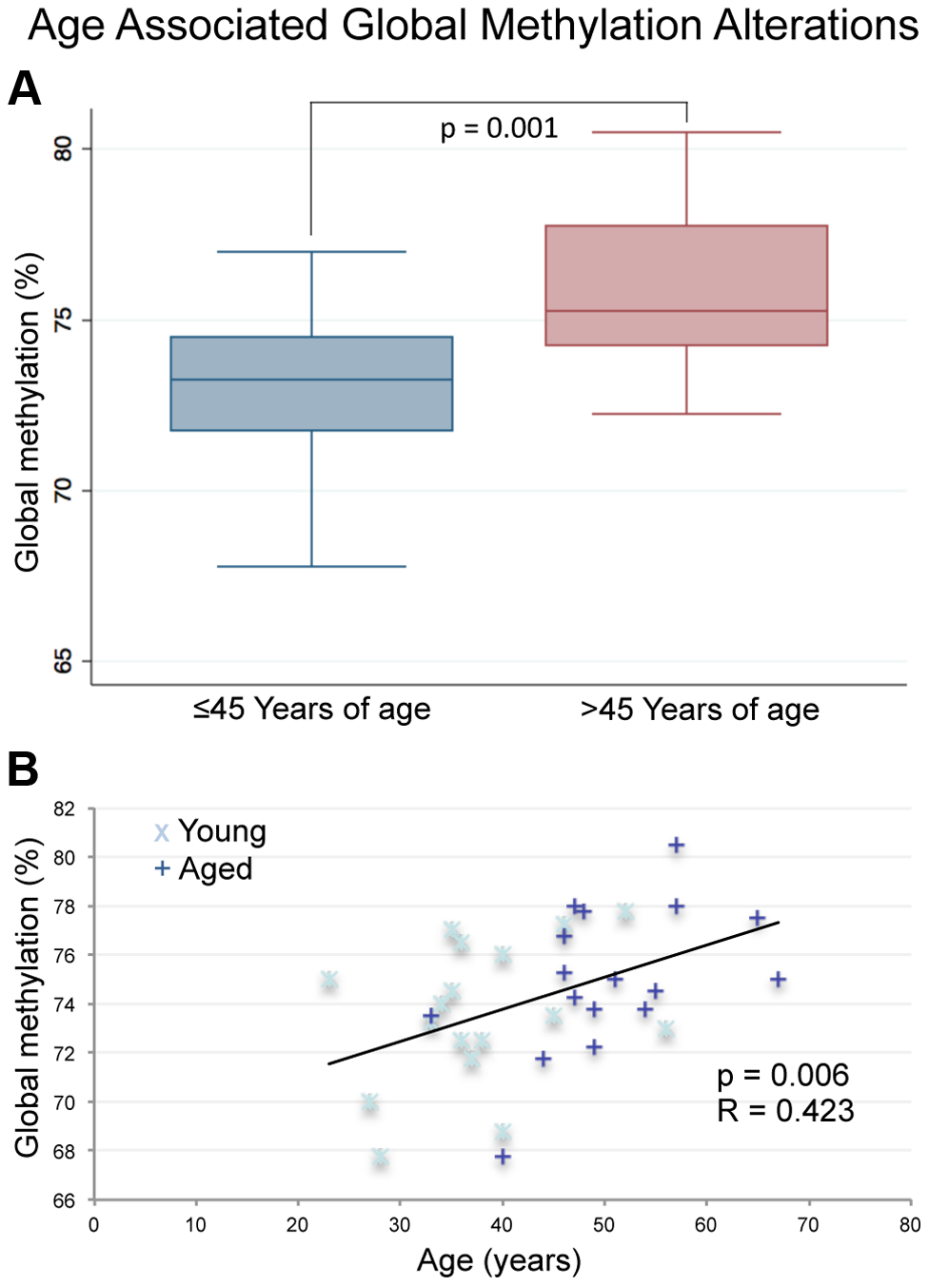
Pyrosequencing results for the LINE-1 global methylation assay. (A) The box plot depicts significantly increased average global methylation with age based on a non-paired t-test of all samples ≤45 (n = 17) years of age vs. all samples >45 (p = 0.001; n = 17). Global methylation was also stratified based only on age at the time of collection for each sample from all 17 donors (a total of 34 samples with each donor represented twice). (B) Linear regression analysis confirmed the significant increases in global sperm DNA methylation with age (p = 0.0062).

To assess global methylation in the samples in question we performed pyrosequencing analysis of long interspersed elements (LINE), a commonly used tool for the analysis of global methylation in many tissues [23], [24]. We identified significant global hypermethylation with age in sperm DNA as previous data from our lab suggests (Figure 1) [25]. Specifically, there was significant hypermethylation with age based on a paired analysis (p = 0.028) or by stratifying the samples by age alone and performing linear regression analysis (p = 0.0062).

### Array analysis

In addition to the global analysis, we performed a high resolution (CpG level) analysis of methylation alterations with age. To perform this we utilized Illumina's Infinium HumanMethylation 450K array. Each sample was hybridized and analyzed on an array and the results were compared to detect changes in methylation that are consistent with age. We utilized a sliding window analysis, coupled with regression analysis (average methylation at identified window versus the age at collection) as an additional filter (any window whose regression p-value was >0.05 was excluded from downstream analysis), to compare changes that are common between paired samples. A Benjamini Hochberg corrected Wilcoxon Signed Rank Test FDR of < = 0.0001 and an absolute log2 ratio > = 0.2 (effectively a change in methylation of approximately 10% or greater) was used as our threshold of significance. Raw FDR values have been transformed for visualization in figures and reference in this text ((−10 log_10_ (*q*-value FDR)), such that a transformed FDR value of 13 = 0.05, 20 = 0.01, 25 = 0.003, 30 = 0.001, and 40 = 0.0001. With this approach we have identified multiple age-associated intra-individual regional methylation alterations that consistently occur within the same genomic windows in most or all of the donors screened. Specifically, we identified a total of 139 regions that are significantly hypomethylated with age (Log_2_ ratio ≤−0.2) and 8 regions that are significantly hypermethylated with age (Log_2_ ratio ≥0.2; Table S1). The average significant window is approximately 887 base pairs in length and contains an average of 5 CpGs with no fewer than 3 in any significant window. Of the 139 hypomethylated regions, 112 are associated with a gene (at either the promoter or the gene body), and of the 8 hypermethylated regions 7 are gene associated. The 8 hypermethylated regions that were found did change in all donor samples, however they did not increase DNA methylation levels beyond 0.1 fraction methylation. In one case we identified 3 significantly hypomethylated windows within a single gene (PTPRN2). Thus there were a total of 110 genes with age-associated hypomethylation.

**Figure 2:**
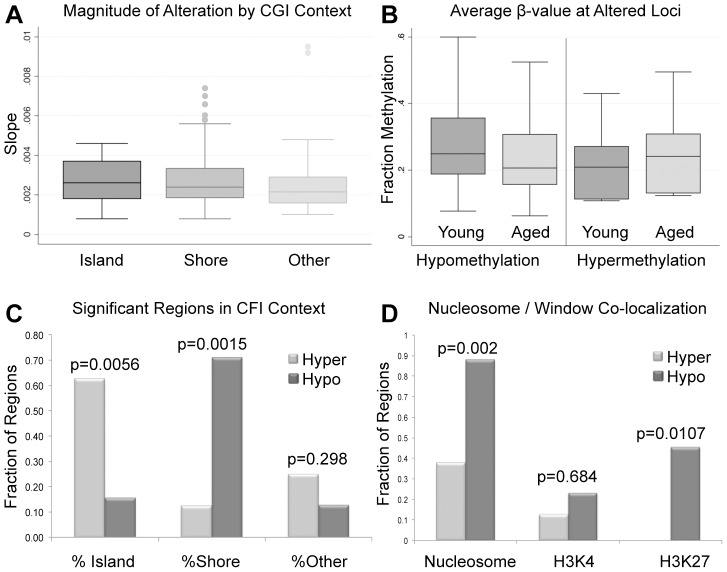
(A) The magnitude of alterations in terms of amount of change per year (reported as slope magnitude) for all regional changes that occur at CpG islands, shores and outside of these regions (other). Average alterations per year were approximately 0.281%. (B) Average β-values for all significant windows (hypomethylation and hypermethylation events) for both aged and young. Average decrease in β-value was approximately 3.9% for intra-individual hypomethylation events and 3.2% for hypermethylation events. (C) the percent of regions of hypermethylation and hypomethylation at CpG islands, shores and outside of these regions (Other). Hypermethylation events were significantly more enriched at islands than were hypomethylation events based on a fisher exact test (p = 0.0056). Hypomethylation events were significantly more enriched at shores in comparison to hypermethylation events (p = 0.0015). Hypermethylation and hypomethylation events were similarly enriched in regions outside of islands and shores. (D) We also investigated the co-localization of nucleosomes (every region of known histone retention) as well as histone modifications (H3K4 methylation, and H3K27 methylation) with our windows of interest. Hypermethylation events were less frequently associated with all retained histones (nucleosomes) or loci with H3K27 methylation when compared to hypomethylation events based on Fisher's Exact Test (p = 0.002; p = 0.0107). Co-localization of hypermethylation or hypomethylation events with H3K4 methylation was statistically similar.

A previous report analyzing multiple somatic tissues suggests that the magnitude of DNA methylation alterations that occurs with age is fairly subtle with an average percent change per year (measured as slope) at a single CpG of approximately 0.05% to 0.15% [19]. Our data, while still subtle, suggest that there may be a stronger effect of age on the methylation alterations in sperm compared with somatic cells. Briefly, in the four tissues screened by Day et al. (blood, brain, kidney and muscle) they identified a total of 8 individual CpGs with a methylation change per year of >0.4% and a single CpG with a yearly change of >0.5%. By comparison, our data have revealed a total of 26 genomic windows (not just individual CpGs) whose average fraction methylation change is >0.4% per year and 13 genomic windows with an average fraction methylation change per year of >0.5% (Figure 2A–B). Specifically in hypermethylated regions, the average fraction methylation change was 0.304% per year (ranging from 0.08% to 0.95% per year). In hypomethylated regions the average fraction methylation change was 0.279% per year (ranging from 0.08% to 0.92% per year). Considering the entire reproductive lifespan of a male, it is not unreasonable to anticipate an average change of 10–12% at these significantly altered regions. Importantly, these alterations all occur in windows with an average initial fraction methylation of <0.6 at the first collection and the majority (67% of altered regions) are also considered to have intermediate methylation based on conventional standards (fraction DNA methylation levels between 0.2 and 0.8; Figure 2B). Despite the increased magnitude of age-associated alterations in sperm when compared to somatic cells these changes are still quite subtle when considering the possible biological impacts at the 119 regions of age-associated alteration that are found at genes (gene bodies, promoters). Gene promoters were defined based on Illumina's array annotation, in general these fall within 1000 bps of the associated gene.

**Figure 3:**
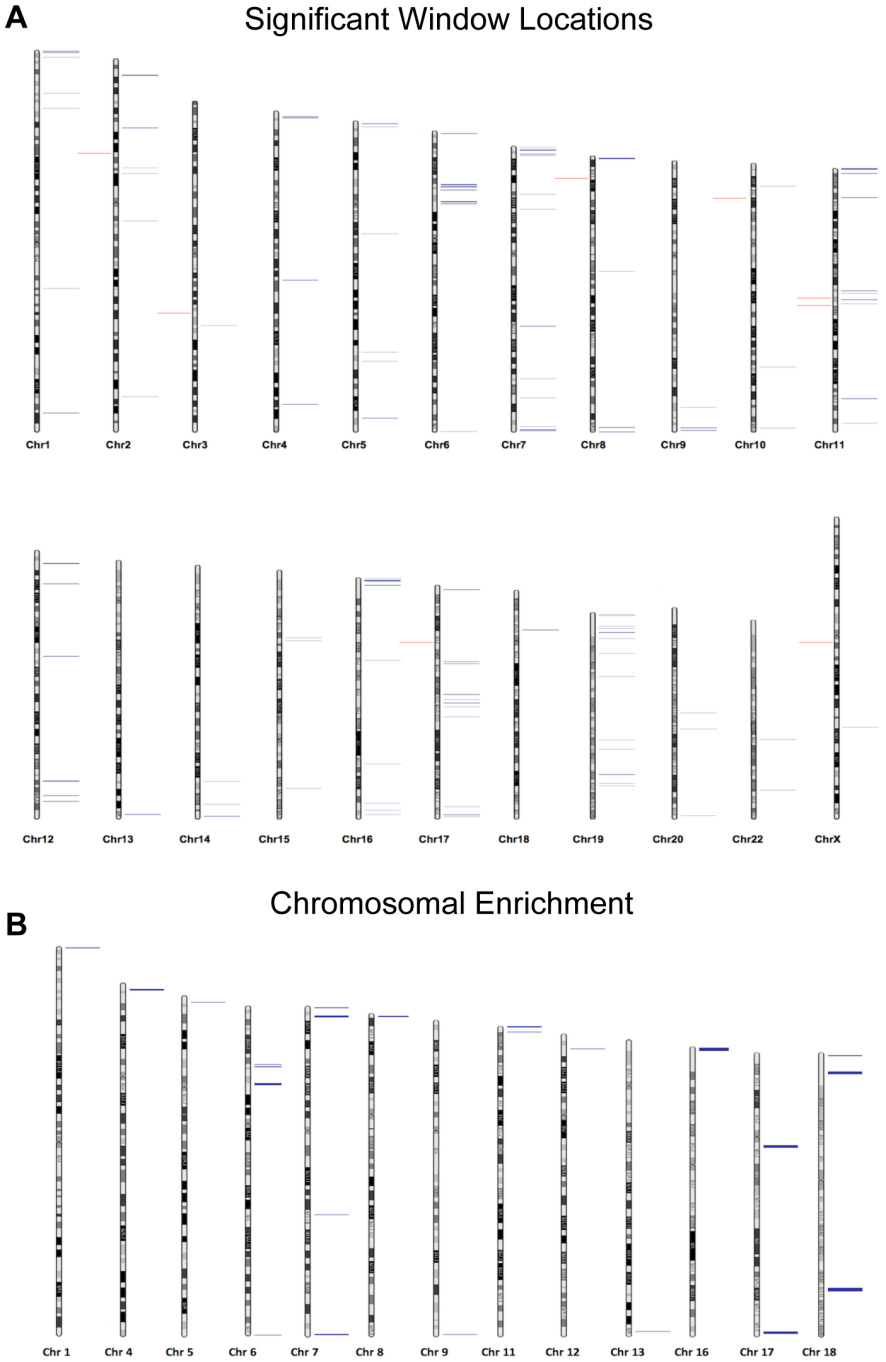
(A) Chromosomal loci of each altered region are depicted where blue marks represent hypomethylation events and red marks hypermethylation events. (B) The Correlation Maps app on the USeq platform was used to locate any specific chromosomal enrichment of altered methylation windows. Specifically, the application called any 100 kb region where at least two significantly altered methylation marks were found. All called chromosomal enrichment regions are displayed though none were found to be significantly enriched over the background.

The significant loci identified in our analyses are located at various genomic features. The majority of regions that undergo age-associated hypomethylation occurred at CpG shores, whereas hypermethylation events are more commonly associated with CpG islands, and these differences are significant in both cases (p = 0.0015 and p = 0.0056 respectively; Figure 2C). It should be noted that while we did observe these significant changes there are slight differences in the baseline fraction methylation at islands and shores between regions with hypomethylation events and those with hypermethylation events (at the highest an absolute fraction methylation change of 0.16). We additionally analyzed the co-localization of windows of age associated methylation alterations with known regions of nucleosome retention in the mature sperm, as well as regions where specific histone modifications are found based on previous work from our laboratory [12]. We found that approximately 88% of regions that are hypomethylated with age are found within 1 kb of known nucleosome retention sites in the mature sperm (Figure 2D). Interestingly, loci that are hypermethylated with age are far less frequently found in regions of histone retention, with only approximately 37.5% being associated with sites where nucleosomes are found, though there are only 8 regions of significance on which to base this analysis. This difference was significant based on a fisher's exact test (p = 0.002). Similarly, 23% of loci with age-associated hypomethylation are associated with H3K4 methylation and 45.3% are associated with H3K27 methylation. The same co-localization is very rare with hypermethylation events (p = 0.0107). Additionally, we analyzed chromosomal enrichment of these marks to determine if there are specific chromosomal regions that are more susceptible to age-related methylation alterations. We found a random distribution of significant age-associated methylation alterations throughout the entire genome with what appears to be enrichment at telomeric and sub-telomeric loci, however this apparent enrichment failed to reach significance (Figure 3).

### Sequencing analysis

**Figure 4:**
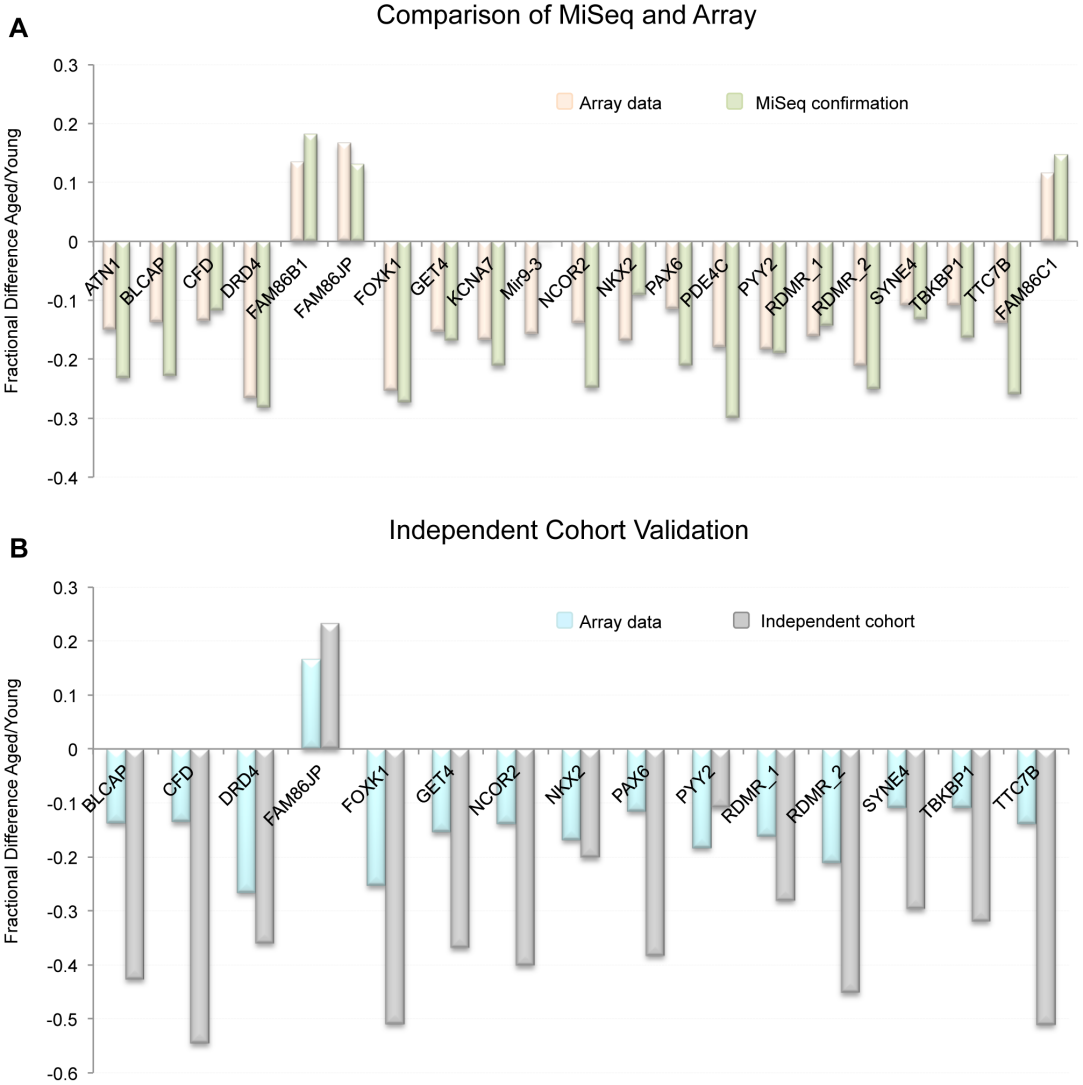
(A) Comparison of MiSeq results to our array results at 21 representative regions. Because beta-values and fraction methylation are generated in a different manner (array vs. sequencing respectively) they are not directly comparable. For this reason we compared the fractional difference for each loci and each technology. This is accomplished by the following equation: fractional difference = (aged value/young value)−1. (B) the fractional difference between young and aged samples at 15 selected loci as measured by array in the 17 donor samples as well as in the independent cohort (19 samples from individuals > = 45 years of age and 47 samples from individuals <25 years of age taken from the general population). On average the fractional difference identified in the independent cohort (as measured by sequencing) was approximately 2.2 times greater in magnitude than was identified in the 17 donors.

To confirm our array data we selected 21 regions found to be significant by our array analysis and subjected them to targeted bisulfite sequencing (on the MiSeq platform) to confirm that the CpGs tiled on the array reflected the entire CpG content within the windows of interest. Specifically, we amplified via PCR, bisulfite converted DNA from each donor (young and aged collections). The PCR was designed to produce amplicons of approximately 300–500 bp that were located within 21 of the regions of significant methylation alteration we identified by array. Our depth of sequencing was quite robust with an average of 2,252 (SE ±371.6) reads per amplicon in each sample. The minimum number of average reads for any one amplicon was 313. In 20 of the 21 gene regions that were analyzed, the array and MiSeq data were similar in both direction and relative magnitude (Figure 4A). In the one case that did not show a similar trend (hypomethylation with age by array and no change by MiSeq) the amplicon was outside the region of the two CpGs that drove the significance of the window. When comparing the methylation of the approximately 300 bp amplicon to the CpG tiled on the array in that same region only, and not the array CpGs over the entire 1000 bp window, the data are in agreement. Taken together, the sequencing run confirmed that our array data is a good representation of the methylation status at all CpGs in our regions of interest.

### Independent cohort analysis at identified regions of interest

To confirm that the sites identified on the array were not only altered in the samples we investigated, but that these loci are also altered with age in the sperm of non-selected individuals in the general population, we have performed an analysis on an independent cohort of individuals from two age groups: young, defined as <25 years of age (n = 47), and aged, defined as ≥45 years of age (n = 19). Average age in the young cohort was 20.46 years of age (SE ±0.18), and in the aged cohort 47.71 years of age (SE ±0.77). We performed a multiplex sequencing run on sperm DNA from these individuals to probe for 15 different regions of interest that were identified with the array data. Briefly, we PCR amplified 15 regions (using bisulfite converted DNA) from each individual (47 young, and 19 aged). The PCR was designed to produce amplicons of approximately 300–500 bp that were located within 15 regions of significant methylation alteration identified by array. Our depth of sequencing was, again, quite robust with approximately 3,645 (SE ±853.4) reads per amplicon in each sample with a minimum average number of reads for any one amplicon of 263. From these data we have confirmed that these genomic regions clearly undergo age-associated methylation alterations (Figure 4B). Interestingly, the average magnitude of alteration is also much higher in our independent cohort than in our initial paired donor sample study (approximately 2.2 times greater on average). This is particularly remarkable when considering that the average age difference in the independent cohort study was 27.2 years, effectively 2.3 times greater than the average age difference of 12.6 years seen in the paired donor analysis. This further supports our regression data in the paired donor study, which generally suggest a linear relationship of methylation alterations with age at most of the identified genomic loci.

### Single molecule analysis of targeted sequencing

**Figure 5:**
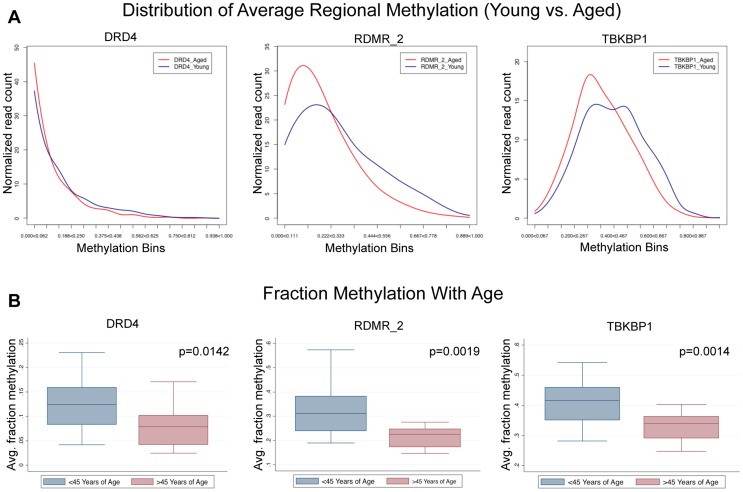
Single molecule analysis reveled 3 distinct alterations that occur with age. (A) DRD4 has only slight alterations associated with age because the young cohort (<45) is strongly hypomethylated initially, and aging simply amplifies this. RDMR_2 is representative of many alterations observed in this analysis which had a strong population shift from moderately hypomethylated to hypomethylated. TBKBP1 is representative of sites that had a bimodal distribution methylation patterns in the young group that becomes stabilized with age. (B) in every case (DRD4, RDMR_2, TBKBP1) each region has significant demethylation with age though the magnitude of change varies.

To address the question of the dynamics of sperm population changes associated with the approximately 0.281% change per year identified in this study we subjected our next generation sequencing data from the paired donor samples to a novel analysis where we compared the sperm population shifts between the young and aged samples. Because the MiSeq platform produces data for each single nucleotide sequence (each representing the methylation status in a single sperm) we are able to determine average methylation at each region for all of the amplicons analyzed. We identified 3 general patterns in methylation profile population shifts that resulted in the age–associated methylation alterations we identified. First, we identified regions whose methylation at an age <45 was strongly hypomethylated, and the methylation profile in individuals >45 years of age is virtually the same, though it is more strongly hypomethylated. In these cases the change is still strikingly significant, but the magnitude of fraction DNA methylation change is minimal. Second, we see a single population in samples collected at <45 years of age that is shifted toward more hypomethylation in samples collected at >45 years of age. Last, we identified a bimodal distribution in samples collected <45 years of age that, in samples >45 years of age, is stabilized into a single population (Figure 5). This could be indicative of at least two sperm subpopulations, which are biased to a single, more hypomethylated sperm population with age. In every case the results suggest that all of the alterations we detected with the array are the result of the entire sperm population being altered in similar subtle ways and not a result of a dramatic alteration in a small portion of the sperm population.

### GO term, Pathway and disease association analysis

The genes affected by the age associated methylation alterations (those that have alterations that occur at their promoter, or gene body) were analyzed by Pathway, GO and disease association analysis. The results indicate that no one GO term or Pathway is significantly altered in our gene group. Similarly, there were no significant diseases or disease classes associated with the genes identified in this study based on results of the disease association tool on DAVID. However the most significant disease hits (those that were significant prior to multiple comparison correction) have both been suggested to have increased incidence in the offspring of older fathers, namely myotonic dystrophy and schizophrenia [2], [7].

**Figure 6:**
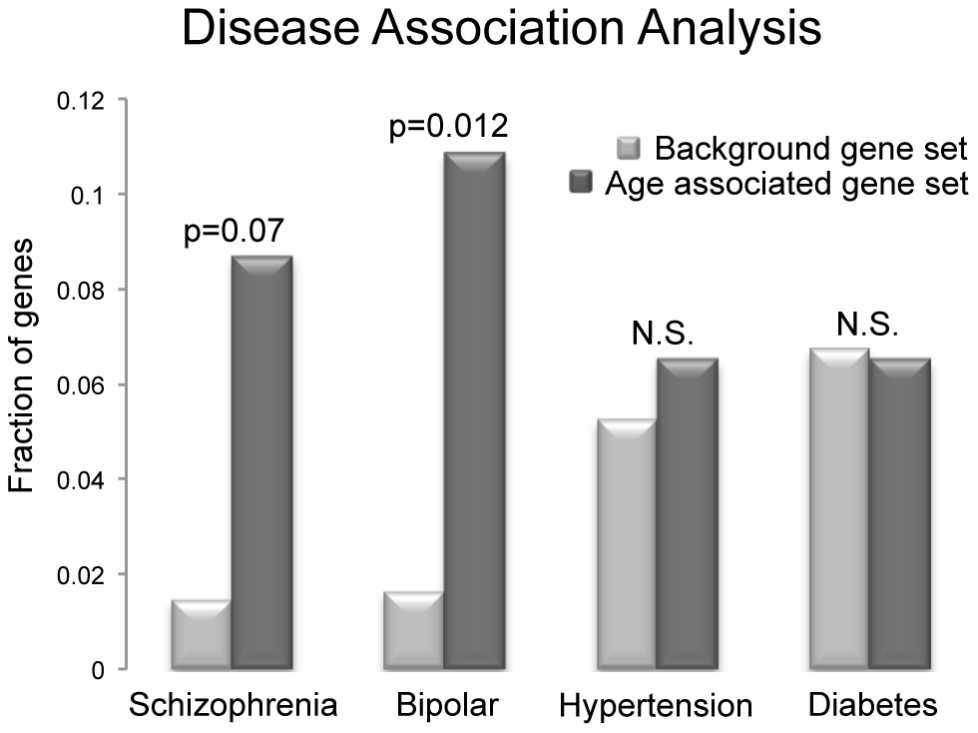
The frequency of disease associations within our gene set was analyzed and compared to the frequency of disease associations for all genes known to be associated with at least a single disease based on GAD annotation. Schizophrenia, bipolar disorder, diabetes mellitus and hypertension were selected, as there were at least 3 genes in our small set of identified genes that are associated with these diseases. Only bipolar disorder was more frequently associated with our identified genes than the background set of genes, based on chi-squared analysis with multiple comparison correction (Bonferonni) of the 117 age associated genes analyzed (p = 0.012), and schizophrenia also trended toward increased frequency (p = 0.07). However, these are not considered significant enrichments if considering all genes in the genome (omitting the filter for a disease connection). The frequency of genes associated with hypertension and diabetes mellitus in the two groups was statistically similar.

To directly investigate the disease association(s) in our set of genes we searched the National Institute of Health's (NIH) genetic association database (GAD). We investigated all 117 genes that were determined to have age associated methylation alterations (110 hypomethylated; 7 hypermethylated) for their various disease associations. From these a total of 46 genes have been confirmed to be associated with either a phenotypic alteration or a disease based on GAD annotation. We identified 4 diseases that were most commonly associated with our set of genes (those disease that are associated with at least 2 genes identified in our study; diabetes mellitus, hypertension, bipolar disorder and schizophrenia). To further investigate these associations, we analyzed the frequency of genes associated with these 4 diseases in our gene set and compared it to their frequency in all 11,306 genes known to be associated with either a phenotypic alteration or a disease. Only bipolar disorder appeared to be more frequently associated with our identified genes than the background set of genes, based on chi-squared analysis with multiple comparison correction (Bonferonni) of the 117 age associated genes identified in our analyses (p = 0.012). Interestingly, schizophrenia also appeared to trend toward increased frequency (p = 0.07; figure 6). However, it is important to note that these are not considered significant enrichments if considering correction for comparisons with all genes in the genome (omitting the filter for a disease connection). The frequency of genetic association between our gene set and the background gene set was statistically similar for both hypertension and diabetes mellitus.

## Discussion

Herein we report alterations to sperm DNA methylation associated with age. Interestingly, our data are in contrast with previous reports of age-associated methylation alterations in somatic cells. Recent literature suggests age-associated global hypomethylation with regional (gene associated) hypermethylation in somatic tissue [20], [21]. In contrast, our data reveal age-associated hypermethylation globally with a strong bias toward hypomethylation regionally. This is less surprising when we take into account the fact that sperm are known to have other age-associated modifications that defy convention (i.e. telomere length) [26]–[28]. Intriguingly, while the methylation alterations reported herein are relatively subtle, they are strikingly significant and are common among individuals at various ages and intervals between collections, suggesting that these regions are consistently altered over time in a linear fashion. Importantly, it appears that many significantly altered regions are at loci that may contribute to various diseases known to have increased incidence in the offspring of older fathers. Coupling these with our data demonstrating that no one GO term or pathway is up or down-regulated in the sperm, as a result of the aging process, suggests that the alterations we observed are the result of regional genomic susceptibility to methylation alteration and not the activation or inhibition of any one cellular program. This hypothesis also comports well with the linear nature of the alterations we observed at most loci. While the nature of this susceptibility is difficult to elucidate, it may be related to chromatin architecture.

It should be noted that while we have identified many intriguing alterations to the sperm methylome, these likely do not represent all of the consistently altered regions that occur in the sperm over time. Our approach was to identify alterations with the use of a “promoter array” which has inherent biases. Specifically, the array is tiled with a higher density of probes at promoter or gene dense regions. Taken together with the use of a restricted window size (1000 bps) for searching the genome, this results in a bias toward identifying regions that are well covered on the array. While this bias is real, it does not invalidate the regions we have identified, but it suggests that more regions may be affected that are poorly covered on the array. Additionally, there are inherent concerns with the collection of tissues over time. One such concern is the difference in freezing methods over the years and the role this may play in methylation profiles. While it is unlikely that this alone could affect methylation profiles, the variances over time should still be reported. Our laboratory's protocol for freezing samples has been consistent for the times of collection of all samples included in this study. We have used the same cryomedium, test yolk buffer, over these years. It is unlikely then, that the methylation patterns in these samples are affected based on any of these variables, and thus the perturbations identified herein represent a true biological change to the sperm epigenome. This is supported by our replication dataset in which young and aged samples were collected concurrently.

### Localization of altered regions

To investigate the attributes of regions that we determined to be most susceptible to methylation alterations, we evaluated the co-localization of significantly altered loci in our study with regions of nucleosome retention in the mature sperm. It appears that hypomethylation events are most commonly associated with sites of nucleosome retention. It should be noted that our criteria for sites of nucleosome retention is simply that our sites of alteration occur within 1 kb of known retention sites and thus there may be a greater degree of complexity in the actual sites of methylation alteration than we have identified. The actual nature of methylation patterns at a higher resolution in these regions (whether the affected regions are flanking or directly associated with histones) is difficult to elucidate due to the nature of array tiling in many of the loci we identified. Interestingly, this same co-localization was not seen with hypermethylation events. Though co-localization patterns are significantly different between the hypomethylation and hypermethylation events, it should be noted that the sample size is quite small in the hypermethylation group (8 significant windows). It should also be noted that while the co-localization of histones and the hypomethylation events we observed in our study are significant, the methylation marks observed are likely established earlier in spermatogenesis and thus may not be affected by the nucleosome architecture in the fully matured sperm. In addition, the alterations identified in this study are not localized everywhere that histones are retained, thus nucleosome retention alone can't be the independent driving force of regional susceptibility to methylation alterations. It should be further noted that our approach was not targeted to observe changes in chromatin co-localization patterns and as such this represents a secondary analysis of these patterns with the use of a “promoter array.” As a result of observing only a selected portion of the genome, there are clear biases that are introduced that should be taken into account when considering these findings.

Recent literature suggests an interesting hypothesis of “selfish spermatogonial selection” that may have application in this study as well [29]. Briefly, the hypothesis states that some gene mutations that are causative of abnormalities in the offspring are beneficial to spermatogenesis and become enriched throughout the aging process in spermatogonial stem cells. Thus, sperm carrying these mutations become more frequent in the population to the detriment of the offspring. Similarly, it is possible that the age-associated methylation alterations we have identified may be in regions that are important to spermatogenesis and thus would be selected for. While the genes identified herein are not well known spermatogenesis hotspots, they may lie close to other genes that are important in development and thus may be subject to a looser chromatin state leaving these genes more susceptible to methylation perturbations.

The hypomethylation events we identified could occur as a result of either active or passive demethylation. For example, regional transcription activity at loci important in spermatogenesis would likely be accompanied by a relaxed chromatin structure that could result in increased frequency of DNA damage over time. Established methylation marks located within this region could then be passively removed through repair mechanisms in the developing sperm. If the removal of this mark is either beneficial or has no effect on spermatogenesis it will persist, and over time similar marks could accumulate at nearby CpGs ultimately leading to the profile we identified in our study. It should also be noted that the accumulation of de novo mutations could lead to a similar profile. It is clear that the number of mutations in the sperm increase with age, and if these mutations involve deamination of cytosine residues the resulting sequence could appear as a loss of methylation with the technologies utilized herein. However, the mutation load, and specifically these C to T transitions, in sperm are stochastic in nature and thus cannot be the primary driving factor for the genomic hotspots of age-associated hypomethylation seen in virtually all of the individuals screened [30]. Alternatively, active enzymatic removal of methylation marks in the sperm might drive age-associated methylation changes. For this to be mechanistically plausible we would have to assume that hypomethylation in the windows we identified is always beneficial to spermatogenesis. While either of these mechanisms is plausible, it is likely that the effects we have identified involve some combination of both.

The mechanics of hypermethylation events with age are more difficult to elucidate, as this, by definition, has to be an active targeted process involving methyltransferase enzymes. However, some evidence from this study indicates DNA sequence may be an important driver of age-related hypermethylation. Of the 7 windows that we identified with gene-associated hypermethylation with age, 4 are associated with the FAM86 family of genes that are categorized not by protein function or genomic location but sequence similarity. This strongly suggests that, at least in part, age associated hypermethylation events at specific loci are driven, either directly or indirectly, by DNA sequence. Interestingly, this family of genes (FAM86) with unknown function has recently been categorized with a larger family of methyltransferase genes, though it remains unclear what the FAM86 target(s) is/are (DNA, Histone, other proteins, etc.). It is important to note that in addition to these regional hypermethyaltion events, globally DNA methylation is markedly increased as well. The possible role of chromatin modifications (histone tail modifications, etc) in this process is also important to note, as what we have identified may be linked to regional histone methylation, acetylation, etc. Such histone modifications may reflect underlying transcriptional changes during spermatogenesis. Taken together, the mechanisms that drive age-related methylation alterations in the sperm remain elusive, but likely involve both active and passive methylation modification.

### Biological significance

It is important to consider two questions in determining the biological impacts of the identified methylation changes in this study. First, are the methylation changes described herein capable of transcriptional alterations? Second, are these methylation changes capable of avoiding embryonic methylation reprogramming? Regardless of the mechanism by which these methylation marks are altered in the sperm over time, it is striking that these changes occur with such consistency between individuals and have such a tight association with age that was seen in both the paired donor analysis and the independent cohort analysis. This is in stark contrast to the relative stability of the sperm methylome seen over time within each individual in the majority of the genome. One limitation of these findings, however, is the magnitude of alterations we have discovered. As described earlier the average fraction methylation alteration per year was approximately a change of 0.281%. Though this seems relatively small, when expanded to include the possible reasonable reproductive years in a male the change would be 10–12%. The increased magnitude of change with increasing age is strongly supported by our independent cohort study where an increase in the age difference between two groups was directly correlated with an increase in the magnitude of methylation alterations at virtually every locus screened in a relatively linear manner. Importantly, based on our analysis of complete nucleotide sequences from our sequencing data it appears that this decrease of 10–12% reflects changes to random CpGs within windows of susceptibility in all sperm, which would manifest in an individual sperm as a mosaically methylated region. The resultant 10–12% change in methylation within every individual sperm (effectively 1 out of every 10 CpGs are demethylated) suggests that every sperm carries similar, more subtle, alterations within these regions on average.

**Figure 7:**
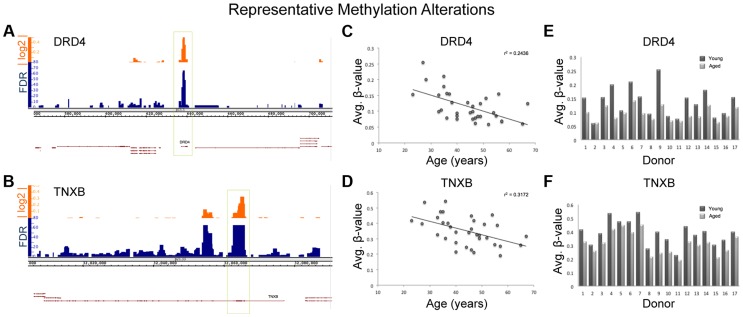
Various descriptive statistics are presented for both TNXB and DRD4; 2 regions of representative methylation alterations. (A,B) The alignment track for each gene is displayed in Integrated Genome Browser (IGB) with the associated false discovery rate (FDR) denoting the significance of the change and the absolute log 2 ratio reflecting the magnitude of the alteration. (C,D) Scatter plots for each sample from all 17 donors (a total of 34 samples with each donor represented twice) with linear regression lines and associated r^2^ values were generated. Regression analysis revealed a significant decrease in methylation with age at both DRD4 and TNXB (p = 0.0005 and p = 0.003 respectively). (E,F) The average methylation within each window (DRD4 and TNXB) was plotted for each paired sample set and is displayed for each donor.

It is important to note that because we only investigated a portion of the regions of interest in our sequencing run (used for confirmation of array results) and the amplicons we probed made up only a portion of the regions of interest, we can not make a definitive overarching statement about the dynamics of methylation profile population shifts in sperm as a result of age. Despite this, the consistency of population shifts in the regions we were able to observe suggests that other regions of interest would likely follow similar patterns. Regardless, the resultant age-associated epigenetic landscape alterations may contribute to disease susceptibility in the offspring despite the small degree of change though the increased risk to the offspring may be relatively small. Figure 7 illustrates the alterations seen at two representative loci from our analysis, Dopamine receptor D4 (DRD4; ENSG00000170956) and tenascin XB (TNXB; ENSG00000168477).

The heritability of such marks is more difficult to elucidate mainly because the current study does not directly address this question. However, this issue needs to be addressed as the identified age-associated methylation alterations in the mature sperm may be removed through the embryonic demethylation wave. Despite the fact that there is no direct evidence of methylation alteration heritability at the specific loci presented in this work, the observed age-associated changes at regions known to be of significance in diseases with increased incidence in the offspring of aged males is striking and warrants further study. The intriguing localization of these alterations suggests that the methylation profile in the mature sperm, at specific loci, either contributes to the increased incidence of associated abnormalities in the offspring or that they reflect (are downstream of) changes that are actually causative of the associated abnormalities in the offspring. Moreover, it has been previously proposed that epigenetic alterations are among the most likely candidates to transmit such transgenerational effects, and we have identified methylation alterations that appear capable of contributing to the various pathologies associated with advanced paternal age. Despite this, future work must still be performed to determine the real impact these marks have on transcription and thus phenotype and disease. Taken together, these subtle yet highly significant, age-associated alterations to the sperm methylation profile are intriguing because of their location and consistency, but more work is required to elucidate the biological impact of these marks.

There are many genes identified in our study that, if biologically affected, may result in pathologies in the offspring. DRD4 is one of the most widely implicated genes in the pathology of both schizophrenia and bipolar disorder as well as many other neuropsychiatric disorders [31], [32]. Interestingly, the entire DRD4 gene itself is hypomethylated with age (Figure 7). TNXB has also been suggested to be associated with schizophrenia based on multiple studies, though the data are controversial [33], [34], and virtually the entire 1^st^ exon of TNXB is hypomethylated with age. Additionally, DMPK (ENSG00000104936), a gene identified in our study, is known to be associated with myotonic dystrophy, a disease for which advanced paternal age is a risk factor [7]. In fact, increases in trinucleotide repeats in DMPK are believed to be the cause of myotonic dystrophy type 1. Importantly, previous data suggests that altered methylation marks may affect trinucleotide instability [35]. These examples represent only a portion of the genes that were identified in our study and support the hypothesis that age-associated DNA methylation alterations in sperm may play a role in the etiology of various diseases associated with advanced paternal age.

### Future directions

There are two important findings in this study. First, that there are any age-associated alterations common among such a varied study population (in terms of the age at collection) is remarkable. Specifically, age-associated methylation alterations occur in the sperm regardless of whether the ages between collections are approximately 20 to 30 years of age or 50 to 60 years of age. Second, the increased frequency of genes associated with bipolar disorder and schizophrenia in our study when compared to all genes associated with disease provides intriguing insight into the increased susceptibility of these specific disorders in the offspring of older fathers. Though frequently hypothesized, this work comprises, to the best of our knowledge, the first direct evidence suggesting the plausibility of epigenetic alterations in the sperm of aged fathers influencing, or even causing, disease in the offspring. Because of the nature of the unique sample set we have utilized in this study future work is needed to directly address a number of questions. Are methylation alterations, similar to those seen in our study, causative of neuropsychiatric disease? Can the methylation marks we observe in our study avoid embryonic demethylation? Future targeted work is required to directly address these questions to enable us to determine the role that these altered methylation marks play in the increased incidence of various diseases seen in the offspring of older fathers.

## Methods

### Ethics statement

The Institutional Review Board at the University of Utah approved this study. Written informed consent was obtained from all participants for their tissues to be utilized for this work.

### Study group

Under an Institutional Review Board approved study our laboratory has accessed samples from 17 sperm donors (of known fertility) that were collected in the 1990's. These donors provided an additional semen sample in 2008, enabling the evaluation of intra-individual changes to sperm DNA methylation with age. These samples are referred to as young (1990's collection) and aged (2008 collection) samples. The age difference between each collection varied between 9 and 19 years, and the age at first collection (“young” sample) was between 23 and 56 years of age.

At every collection donors were required to strictly follow the University of Utah Andrology Laboratory collection instructions, which includes abstinence time of between 2 and 5 days. The whole ejaculate (no sperm selection method was employed) collected at each visit was frozen in a 1∶1 ratio with Test Yolk Buffer (TYB; Irvine Scientific, Irvine, CA) and stored in liquid nitrogen prior to DNA isolation. Samples were thawed and the DNA was extracted simultaneously to decrease batch effects. Sperm DNA was extracted with the use of a sperm-specific extraction protocol used routinely in our laboratory [36]. Briefly, sperm DNA was isolated by enzymatic and detergent-based lysis followed by treatment with RNase and finally DNA precipitation using isopropanol and salt, with subsequent DNA cleanup using ethanol. To ensure the absence of potential contamination from somatic cells the samples were visually inspected prior to DNA extraction. Additionally, we analyzed our sequencing results in an attempt to identify reads that did not match the methylation profile of sperm but instead reflected that of leukocytes. We also analyzed imprinted regions from our array data in an attempt to identify fraction methylation values that were inconsistent with previous reports of sperm DNA methylation patterns. Specifically, at a region of the IGF-2 locus that is tiled on the 450K array, it has been previously shown that sperm DNA is strongly hypermethlyated with a fraction methylation of approximately 0.8–0.85 and in leukocytes this same region is strongly demethylated with a fraction methylation of <0.1 [37]. Our array data indicate average methylation in every sample screened at these sites is approximately 0.844. In summary, with neither approach did we identify any signal that indicated leukocyte or other somatic cell contamination.

### Pyrosequencing analysis

Each sample was subjected to pyrosequencing analysis of a portion of the LINE-1 repetitive element for the purpose of confirming previously determined global methylation changes with age. Briefly, isolated sperm DNA samples were submitted to EpigenDx (Hopkinton, MA) for pyrosequencing analysis. Qiagen's PyroMark LINE1 kit was used to determine methylation status at each CpG investigated with the assay. The experiment was performed based on manufacturer recommendations. The resultant values for each CpG are reported as fraction methylation, or the percent of methylated cytosines at that specifc CpG position. The average of these values was calculated for each individual (young and aged), and the values were compared both by linear regression and by a paired t-test.

### Microarray analysis

Each of the paired samples for the 17 donors (a total of 34 samples) was subjected to array analysis using the Infinium HumanMethylation 450 Bead Chip micro-array (Illumina, San Diego CA). Extracted sperm DNA was bisulfite converted with EZ-96 DNA Methylation-Gold kit (Zymo Research, Irvine CA) according to manufacturer's recommendations. Converted DNA was then hybridized to the array and analyzed according to Illumina protocols at the University of Utah genomics core facility. Once scanned and analyzed for methylation levels at each CpG a β-value was generated by applying the average methylated and unmethylated intensities at each CpG using the calculation: β-value = methylated/(methylated+unmethylated). The resultant β-value ranges from 0 to 1 and indicates the relative levels of methylation at each CpG with highly methylated sites scoring close to 1 and unmethylated sites scoring close to 0.

The raw data were subjected to normalization to ensure the removal of poorly performing probes from the downstream analysis (probes with a QC p<0.05). Batch effect correction and basic descriptive analyses of the microarray data were performed using Partek (St. Louis MO). More in depth analysis was performed using the USeq platform with the application Methylation Array Scanner which identifies regions of altered methylation that are common among individuals utilizing a sliding window analysis. Briefly, paired data from each donor (young and aged) was subjected to a 1000 base pair sliding window analysis where regions of altered methylation with age that are common among donors were called by Wilcoxon Signed Rank Test. To diminish the influence of outliers in the data set, methylation for a specific window was reported as a pseudo-median and differences between the young and aged sample are reported as log 2 ratios. Two thresholds were applied to identify windows with significant differential methylation. A Benjamini Hochberg corrected Wilcoxon Signed Rank Test FDR of < = 0.0001 (> = transformed FDR of 40) and an absolute log2 ratio > = 0.2 was used as our threshold for significance. Raw FDR values were transformed for visualization in figures and reference in this text ((−10 log_10_ (*q*-value FDR)), such that a transformed FDR value of 13 = 0.05, 20 = 0.01, 25 = 0.003, 30 = 0.001, and 40 = 0.0001, etc. We selected this robust level of significance, as opposed to an FDR of > = 13 (corrected p-value of 0.05), to ensure that we selected only the most striking alterations that are consistently perturbed in most or all of the individuals screened. To confirm the significance of each of the called windows we subjected the mean β-value within the window for each donor (young and aged samples) to a paired t-test. Following this initial filter we additionally subjected each significant window to a regression analysis (age at time of collection versus average methylation within significant windows) to determine the relationship between age and mean methylation within each window. Regression analysis and paired t-tests were performed using STATA 11 software package. A p-value of <0.05 was considered significant for these analyses.

### Sequencing analysis

We performed multiplex sequencing in a replication cohort as a confirmation that the alterations identified in the paired donors via array represent methylation alterations that are common in human sperm with age.

First, each donor sample used in the array study was additionally subjected to targeted bisulfite sequencing at loci determined to be most consistently altered based on the window analysis. This step was designed to confirm the array results and to provide greater depth of coverage of the CpGs in the windows of interest. Primers for 21 loci were designed using MethPrimer (Li Lab, UCSF). PCR was performed on samples following sperm DNA bisulfite conversion with EZ-96 DNA Methylation-Gold kit (Zymo Research, Irvine CA). PCR products were purified with QIAquick PCR Purification Kit (Qiagen, Valencia CA) and were pooled for each sample. The pooled products were delivered to the Microarray and Genomic Analysis core facility at the University of Utah for library prep which included shearing of the DNA with a Covaris sonicator to generate products of approximately 300 base pairs, in preparation for 150 bp paired end sequencing, and the addition of sample-specific barcodes for all 34 samples. Multiplex sequencing was then performed on a single lane on the MiSeq platform (Illumina, San Diego CA).

Second, 19 sperm DNA samples from an independent, unselected cohort of general population donors who were ≥45 of ages were selected and compared to 47 sperm DNA samples from general population donors who were <25 years of age. These samples underwent the same preparation as described above for multiplex sequencing, though only 15 amplicons were targeted in this study of larger sample size. Average fraction methylation for each window was determined and was subjected to unpaired t tests between the young and aged groups.

### Single molecule analysis of targeted bisulfite sequencing data

Bisulfite sequencing data was aligned against the human reference genome Hg19 using Novoalign. The aligned reads were processed using Novoalign Bisulfite Parser, BisStat and Parse Point Data Context for CG from the USeq package. The binned CpG graphs were generated using a modified version of the Allelic Methylation Detector from the USeq package. In short, all reads were queried for their number of CpGs. A consensus CpG number was then taken based on the highest number of CpGs per read and a minimum of 10% of all aligned reads (approximately 100 reads per region) must cover said number of CpGs. The consensus CpG number then served as the basis for the number of bins per region. Samples that were donated at an age of 45 years or older were coalesced *in silico* in the “aged donor group”. Conversely, samples younger than 45 years were grouped in the “young donor group”. All reads for the consensus CpG count were summed up based on their age group and then normalized to a 100 reads total. The graphs plotting normalized reads to methylation bins were then generated using the spline function from the R package.

### GO term/Pathway/disease association analysis

GO term Analysis was performed with Gene Ontology Enrichment Analysis and Visualization Tool (GOrilla; cbl-gorilla.cs.technion.ac.il). Pathway and disease association analysis was performed on the Database of Annotation, Visualization, and Integrated Discovery (DAVID; david.abcc.ncifcrf.gov) v6.7. Additional disease association analysis was performed directly on the National Institute of Health's Genetic Association Database (GAD; geneticassociationdb.nih.gov).

### Additional statistical analyses

Fishers exact test was used to determine the differences in frequencies of genes associated with particular diseases between our significant gene group and a background group. This analysis was also used to detect the differences in frequencies of windows that were found in regions of histone retention in the hypomethylation group and the hypermethylation group. Additionally, regression analysis was utilized to determine relationships between age and methylation status at various loci. STATA software package was used to test for significance with a p<0.05 being considered a significant finding.

## Supporting Information

Table S1 Genomic features of significantly altered windows. Represented in this table are the windows of significance that were identified in our study as well as their transformed FDR, log 2 ratio, association to genes, association to known DMR, and CpG Island context.
(DOCX)
