# Supplementary material for: Age-Associated Sperm DNA Methylation Alterations: Possible Implications in Offspring Disease Susceptibility
Source: PLoS Genet. 2014 Jul 10;10(7):e1004458. doi: 10.1371/journal.pgen.1004458 (PMC4091790; doi:10.1371/journal.pgen.1004458)
Supplement: Table S1 — Genomic features of significantly altered windows. Represented in this table are the windows of significance that were identified in our study as well as their transformed FDR, log 2 ratio, association to genes, association to known DMR, and CpG Island context. (DOCX) [file pgen.1004458.s001.docx]

Table S1: Genomic Features of Significantly Altered Windows

| **Gene** | **Genomic Feature** | **CpG Island Association** | **DMR** | **Log2 Ratio** | **FDR** | **r squared** |
| --- | --- | --- | --- | --- | --- | --- |
| ARC | Gene Body | North Shore | N/A | -0.2433 | 65.69 | 0.1902 |
| ATHL1 | Gene Body | Island/South Shore | N/A | -0.2932 | 65.69 | 0.1714 |
| ATN1 | Promoter | North Shelf | N/A | -0.3702 | 65.69 | 0.4421 |
| ATXN7L3 | Promoter | North Shore | N/A | -0.2158 | 65.69 | 0.3413 |
| BEGAIN | Promoter | South Shore | N/A | -0.2747 | 65.69 | 0.4085 |
| BLCAP | Gene Body | North Shore | N/A | -0.2366 | 65.69 | 0.4881 |
| C1orf122 | Promoter | North Shore | N/A | -0.2272 | 65.69 | 0.5157 |
| C6orf48 | Gene Body | South Shore | N/A | -0.2061 | 65.69 | 0.1544 |
| CCDC114 | Promoter | North Shore | N/A | -0.3703 | 65.69 | 0.5512 |
| CCDC144NL | Promoter / Gene Body | Island | N/A | 0.2034 | 65.69 | 0.1989 |
| CFD | Promoter | North Shore | N/A | -0.2795 | 65.69 | 0.3099 |
| CLIC1 | Gene Body | South Shore | N/A | -0.2159 | 65.69 | 0.2098 |
| CNN1 | Promoter / Gene Body | N/A | N/A | -0.2591 | 65.69 | 0.2501 |
| CNTNAP1 | Promoter | North Shore | RDMR | -0.2157 | 65.69 | 0.3904 |
| DLL1 | Gene Body | Island/North Shore | N/A | -0.2937 | 65.69 | 0.1544 |
| DOK1 | Promoter | North Shore | CDMR | -0.2528 | 65.69 | 0.4926 |
| DRD4 | Gene Body | Island | N/A | -0.5705 | 65.69 | 0.3172 |
| EFCAB4A | Gene Body | Island | N/A | -0.3166 | 65.69 | 0.2888 |
| ELANE | Promoter / Gene Body | North Shore | N/A | -0.5163 | 65.69 | 0.1359 |
| GAPDH | Promoter | North shore | RDMR | -0.2191 | 65.69 | 0.2135 |
| GET4 | Promoter | Island/North Shore | N/A | -0.2080 | 65.69 | 0.316 |
| GPANK1 | Gene Body | North Shore | RDMR | -0.2451 | 65.69 | 0.3234 |
| GPR45 | Promoter / Gene Body | Island/North Shore | N/A | -0.2399 | 65.69 | 0.3908 |
| KCNF1 | Gene Body | Island | N/A | -0.3344 | 65.69 | 0.1838 |
| KCNQ1 | Gene Body | Island/North Shore | N/A | -0.2991 | 65.69 | 0.2046 |
| LOC154449 | Promoter | North Shelf | N/A | -0.2064 | 65.69 | 0.122 |
| MIR22HG | Gene Body | North Shore | N/A | -0.2347 | 65.69 | 0.2404 |
| MPPED1 | Gene Body | Island | N/A | -0.2851 | 65.69 | 0.1553 |
| N/A | N/A | HMM Island | N/A | -0.2041 | 65.69 | 0.2629 |
| N/A | N/A | Island/North Shore | N/A | -0.2363 | 65.69 | 0.3355 |
| N/A | N/A | North Shore | N/A | -0.3082 | 65.69 | 0.2066 |
| N/A | N/A | Island/North Shore | N/A | -0.3820 | 65.69 | 0.1795 |
| PCOLCE | Promoter / Gene Body | North Shore | N/A | -0.2438 | 65.69 | 0.1543 |
| PITPNM1 | Promoter | North Shore | N/A | -0.2669 | 65.69 | 0.4935 |
| PPP1R18 | Gene Body | Island/North Shore | N/A | -0.2754 | 65.69 | 0.3867 |
| PRSS22 | Promoter | South Shore | N/A | -0.2486 | 65.69 | 0.5034 |
| PYY2 | Promoter / Gene Body | North Shore | N/A | -0.3241 | 65.69 | 0.6317 |
| SECTM1 | Gene Body | Island | N/A | -0.2568 | 65.69 | 0.3782 |
| SYNE4 | Promoter | North Shore | N/A | -0.2383 | 65.69 | 0.5805 |
| TBKBP1 | Gene Body | Island | N/A | -0.2449 | 65.69 | 0.4863 |
| THBS3 | Promoter / Gene Body | North Shore | N/A | -0.2657 | 65.69 | 0.5953 |
| TNXB | Gene Body | Island | N/A | -0.3319 | 65.69 | 0.2436 |
| UTS2R | Promoter / Gene Body | Island/North Shore | N/A | -0.2767 | 65.69 | 0.2616 |
| ZNF358 | Promoter / Gene Body | Island/North Shore | N/A | -0.2473 | 65.69 | 0.1876 |
| KDM2B | Promoter | South Shore | RDMR | -0.3003 | 65.67 | 0.241 |
| NSG1 | Promoter | North Shore | N/A | -0.2899 | 65.47 | 0.5232 |
| SEZ6 | Gene Body | Island/North Shore | N/A | -0.4530 | 65.05 | 0.43 |
| LMO3 | Promoter | N/A | N/A | -0.3627 | 64.24 | 0.2074 |
| HOXA10 | Promoter / Gene Body | Island/North Shore | N/A | -0.2148 | 64.21 | 0.3354 |
| DAPK3 | Promoter | North Shore | RDMR | -0.3932 | 63.18 | 0.3728 |
| N/A | N/A | Island/North Shore | N/A | -0.3281 | 62.21 | 0.2824 |
| N/A | N/A | South Shore | N/A | -0.2993 | 62.03 | 0.125 |
| NSMF | Gene Body | Island/North Shore | N/A | -0.2249 | 61.30 | 0.329 |
| TOR4A | Promoter | Island/North Shore | N/A | -0.3046 | 61.09 | 0.3998 |
| LDLRAD4 | Promoter | N/A | N/A | -0.2502 | 60.61 | 0.264 |
| N/A | N/A | North Shore | RDMR | -0.2866 | 58.83 | 0.5618 |
| PTPRN2_3 | Gene Body | North Shore | N/A | -0.2391 | 58.31 | 0.151 |
| SSTR5 | Gene Body | Island/North Shore | N/A | -0.2381 | 57.88 | 0.1457 |
| LOC134368 | Gene Body | South Shore | RDMR | -0.2695 | 57.71 | 0.292 |
| GRB7 | Promoter | N/A | N/A | -0.2087 | 57.48 | 0.3144 |
| GNB2 | Gene Body | South Shore | N/A | -0.2238 | 57.45 | 0.1312 |
| SNHG1 | Promoter | North Shore | N/A | -0.2004 | 57.39 | 0.404 |
| LOC653566 | Promoter | N/A | N/A | -0.2929 | 56.31 | 0.2672 |
| N/A | N/A | HMM Island | N/A | -0.2479 | 56.06 | 0.1969 |
| PDE4C | Gene Body | Island/South Shore | N/A | -0.2858 | 55.53 | 0.4673 |
| DLGAP2 | Gene Body | Island/North Shore | N/A | -0.2109 | 55.49 | 0.1296 |
| MRPL36 | Gene Body | North Shore | N/A | -0.2268 | 55.34 | 0.3998 |
| NCOR2 | N/A | HMM Island | N/A | -0.2106 | 55.34 | 0.583 |
| N/A | N/A | North Shore | CDMR | -0.2107 | 54.57 | 0.1157 |
| N/A | N/A | N/A | CDMR | -0.2813 | 52.81 | 0.2763 |
| KCNA7 | Promoter | South Shore | N/A | -0.3664 | 52.24 | 0.5066 |
| CACNA1H | Gene Body | South Shore | N/A | -0.2855 | 51.96 | 0.1695 |
| IRS4 | Gene Body | North Shore | RDMR/CDMR | -0.2273 | 51.23 | 0.2364 |
| KRT19 | Promoter | South Shore | N/A | -0.2701 | 51.08 | 0.3463 |
| LRFN2 | Gene Body | North Shore | RDMR | -0.2525 | 51.08 | 0.2967 |
| WFDC1 | Gene Body | Island | N/A | -0.2966 | 50.49 | 0.2675 |
| APBA2 | Promoter | N/A | N/A | -0.3989 | 50.10 | 0.3216 |
| USP36 | Gene Body | North Shore | RDMR | -0.3108 | 49.92 | 0.2693 |
| PAX2 | Gene Body | South Shore | N/A | -0.3545 | 49.15 | 0.2825 |
| PTPRN2_1 | Gene Body | North Shore | N/A | -0.2828 | 48.41 | 0.3052 |
| N/A | N/A | North Shore | RDMR | -0.2138 | 47.98 | 0.4739 |
| N/A | N/A | HMM Island | N/A | -0.2144 | 47.75 | 0.2672 |
| UNKL | Promoter / Gene Body | Island/North Shore | N/A | -0.2483 | 47.55 | 0.4327 |
| FAM86JP | Promoter | Island/North Shore | N/A | 0.2012 | 47.43 | 0.2884 |
| TTC7B | Promoter | South Shore | N/A | -0.2192 | 47.25 | 0.5194 |
| FAM86C2P | Promoter / Gene Body | Island | N/A | 0.2310 | 46.89 | 0.2156 |
| GRIN1 | Gene Body | Island/North Shore | N/A | -0.3017 | 46.65 | 0.2898 |
| LFNG | Gene Body | South Shore | N/A | -0.3641 | 46.65 | 0.1898 |
| N/A | N/A | HMM Island | N/A | 0.2835 | 46.65 | 0.3944 |
| N/A | N/A | North Shore | RDMR | -0.3885 | 46.65 | 0.5595 |
| SOHLH1 | Promoter / Gene Body | Island/North Shore | N/A | -0.2081 | 46.39 | 0.1542 |
| N/A | N/A | South Shore | RDMR | -0.3423 | 46.34 | 0.1679 |
| N/A | N/A | Island/North Shore | N/A | -0.2100 | 46.34 | 0.3924 |
| SLC22A18AS | Gene Body | South Shore | N/A | -0.2397 | 46.34 | 0.5081 |
| PURA | Promoter | Island/North Shore | N/A | -0.2042 | 46.08 | 0.4237 |
| NFAT5 | Promoter | North Shore | RDMR | -0.2129 | 46.05 | 0.1748 |
| DMPK | Gene Body | Island | N/A | -0.3335 | 46.04 | 0.2442 |
| LOC100133461 | Promoter | North Shelf | N/A | -0.4967 | 46.04 | 0.3899 |
| N/A | N/A | Island/North Shore | CDMR | -0.2369 | 46.04 | 0.4311 |
| N/A | N/A | HMM Island | N/A | -0.3640 | 46.04 | 0.2529 |
| PTPRN2_2 | Gene Body | Island/North Shore | N/A | -0.2666 | 46.04 | 0.1169 |
| PITX1 | Gene Body | North Shore | CDMR | -0.2952 | 45.96 | 0.1888 |
| ARHGEF10 | Gene Body | N/A | N/A | -0.3564 | 45.72 | 0.2585 |
| N/A | N/A | North Shore | N/A | -0.7087 | 45.72 | 0.222 |
| PALM | Gene Body | Island | N/A | -0.2109 | 45.72 | 0.3631 |
| C7orf50 | Gene Body | North Shore | N/A | -0.2133 | 45.54 | 0.1568 |
| SEMA6B | Gene Body | Island/North Shore | CDMR | -0.3163 | 45.39 | 0.3574 |
| FOXK1 | Gene Body | South Shore | RDMR | -0.4457 | 45.27 | 0.4838 |
| FAM86C1 | Promoter / Gene Body | Island | N/A | 0.2260 | 45.18 | 0.1453 |
| ADAMTS8 | Promoter | South Shore | N/A | -0.2193 | 44.74 | 0.5308 |
| N/A | N/A | North Shore | N/A | -0.2771 | 44.67 | 0.2686 |
| EDARADD | Promoter | North Shore | N/A | -0.2506 | 44.52 | 0.3686 |
| FAM86B2 | Promoter | Island | N/A | 0.2238 | 44.48 | 0.2209 |
| AGRN | Promoter | South Shore | N/A | -0.5087 | 44.46 | 0.3049 |
| LEMD2 | Promoter | North Shore | N/A | -0.2055 | 44.46 | 0.414 |
| MTMR8 | Promoter / Gene Body | Island/North Shore | N/A | 0.2070 | 44.27 | 0.3698 |
| MIR9-3 | Promoter | Island/North Shore | N/A | -0.2235 | 44.17 | 0.4838 |
| KRT7 | Promoter | North shore | N/A | -0.2041 | 44.15 | 0.276 |
| NKX2 | Promoter | Island/North Shore | RDMR | -0.3287 | 44.01 | 0.3185 |
| N/A | N/A | North Shore | N/A | -0.2408 | 43.86 | 0.3225 |
| N/A | N/A | North Shore | RDMR | -0.3785 | 43.86 | 0.6517 |
| N/A | N/A | North Shore | RDMR | -0.3876 | 43.56 | 0.3218 |
| USP6NL | Gene Body | Island | N/A | -0.4037 | 43.54 | 0.1384 |
| N/A | Promoter | North Shore | N/A | -0.2067 | 43.22 | 0.3973 |
| N/A | N/A | Island | N/A | -0.2748 | 42.66 | 0.5203 |
| NBLA00301 | Gene Body | North Shore | RDMR | -0.2964 | 42.35 | 0.5779 |
| AJAP1 | Gene Body | South Shore | RDMR | -0.3908 | 42.06 | 0.1215 |
| CRYBA2 | Gene Body | North Shore | N/A | -0.2093 | 42.06 | 0.587 |
| CTF1 | Promoter | South Shore | N/A | -0.2488 | 42.06 | 0.501 |
| FOXF2 | Gene Body | South Shore | RDMR/CDMR | -0.2036 | 41.96 | 0.3976 |
| MAP4K1 | Promoter | North Shore | N/A | -0.2117 | 41.91 | 0.3082 |
| N/A | N/A | HMM Island | N/A | -0.2422 | 41.86 | 0.2107 |
| BCL11A | Gene Body | N/A | N/A | 0.2415 | 41.79 | 0.2955 |
| N/A | N/A | North Shore | RDMR | -0.2307 | 41.76 | 0.529 |
| LONP1 | Gene Body | Island | N/A | -0.2769 | 41.19 | 0.3134 |
| N/A | N/A | HMM Island | N/A | -0.2885 | 41.19 | 0.3396 |
| TBC1D10A | Gene Body | North Shore | N/A | -0.3085 | 41.19 | 0.528 |
| CALCA | Gene Body | North Shore | N/A | -0.2781 | 40.89 | 0.2362 |
| DNMT3B | Gene Body | South Shore | RDMR | -0.3683 | 40.89 | 0.2687 |
| VAX2 | Gene Body | North Shore | RDMR | -0.2485 | 40.89 | 0.3199 |
| ZFPM1 | Gene Body | Island | N/A | -0.2848 | 40.76 | 0.1458 |
| OXLD1 | Gene Body | North Shore | N/A | -0.2737 | 40.60 | 0.3644 |
| FSCN1 | Gene Body | South Shore | RDMR | -0.3639 | 40.31 | 0.3546 |
| FXYD6 | Promoter | South Shore | N/A | -0.3141 | 40.31 | 0.2952 |
| NADK | Promoter | South Shore | RDMR | -0.2196 | 40.31 | 0.3951 |
| PARP12 | Gene Body | North Shore | CDMR | -0.2035 | 40.31 | 0.3821 |
| TBX5 | Promoter / Gene Body | Island/North Shore | N/A | -0.2904 | 40.13 | 0.3641 |
